# A Refunding Scheme to Incentivize Narrow-Spectrum Antibiotic Development

**DOI:** 10.1007/s11538-022-01013-7

**Published:** 2022-04-22

**Authors:** Lucas Böttcher, Hans Gersbach

**Affiliations:** 1 Computational Social Science, Frankfurt School of Finance and Management, 60322 Frankfurt am Main, Germany; 2 Department of Computational Medicine, UCLA, Los Angeles, 90095-1766 USA; 3 Center of Economic Research at ETH Zurich and CEPR, 8092 Zurich, Switzerland

**Keywords:** Antibiotic resistance dynamics, Antibiotics dilemma, RD incentives, Refunding scheme, Narrow-spectrum antibiotics, RD uncertainty

## Abstract

The rapid rise of antibiotic resistance is a serious threat to global public health. The situation is exacerbated by the “antibiotics dilemma”: Developing narrow-spectrum antibiotics against resistant bacteria is most beneficial for society, but least attractive for companies, since their usage and sales volumes are more limited than for broad-spectrum drugs. After developing a general mathematical framework for the study of antibiotic resistance dynamics with an arbitrary number of antibiotics, we identify efficient treatment protocols. Then, we introduce a market-based refunding scheme that incentivizes pharmaceutical companies to develop new antibiotics against resistant bacteria and, in particular, narrow-spectrum antibiotics that target specific bacterial strains. We illustrate how such a refunding scheme can solve the antibiotics dilemma and cope with various sources of uncertainty that impede antibiotic R &D. Finally, connecting our refunding approach to the recently established Antimicrobial Resistance (AMR) Action Fund, we discuss how our proposed incentivization scheme could be financed.

## Introduction

*The Medical Problem*. According to the World Health Organization (WHO), antibiotic resistance is a serious threat to global public health (see, e.g., the WHO antimicrobial resistance factsheet 2019). Some studies see in the emergence of antimicrobial resistance (AMR) (Coburn 2021) the beginning of a postantibiotic era and a societal challenge that some researchers compare to the one posed by climate change (Laxminarayan 2013). In the European Union, more than 33,000 people die every year due to infections caused by drug-resistant microbes. The associated yearly AMR-related healthcare costs and productivity losses are estimated to be more than 1.5 billion Euros (Anderson et al. 2019). A recent study (Murray et al. 2022) found that the global AMR death toll in 2019 was at least about 1 million.

Antibiotic resistance results from mutations in microbes and from evolutionary pressure, which selects those mutations that are resistant against certain antibiotics.^1^ The large-scale use of antibiotics in medical and agricultural settings in high-income countries led to the emergence of various multi-resistant bacterial strains. Recent findings indicate that certain strains of Enterobacteriaceae even developed resistances against the usually highly effective class of carbapenems (Jacob 2013). Carbapenems are so-called *drugs of last resort*, only used if other antibiotic agents fail to stop the proliferation of microbes.

The reasons for the emergence of antibiotic resistance and the decline in effective treatment possibilities are complex, but a major conclusion from the medical literature is that the use of narrow-spectrum antibiotics may lead to a slower development of antibiotic resistance (Gould and van der Meer 2011; May 2006; De Man et al. 2000; Dortch 2011; Maxson and Mitchell 2016). A “narrow-spectrum” antibiotic only affects one strain or a small number of bacterial strains when given to a patient. New narrow-spectrum antibiotics against particular resistant bacteria would thus be highly effective in slowing down antibiotic resistance.

Treatment protocols involving narrow-spectrum antibiotics have been implemented by some northern-European countries such as Norway and Sweden (Torfoss et al. 2012; Mölstad 2008). The Norwegian strategy is based on penicillin G and aminoglycoside as initial treatment substances (Torfoss et al. 2012), and it avoids broad-spectrum $\beta$-lactam antibiotics.

*The Economic and Business Problem*. The use of an antibiotic against bacterial infections entails two economic problems. First, the use of antibiotics exerts a negative externality on all individuals, due to the possible emergence of resistant bacteria and the associated risks for global public health. The antibiotic resistance problem has thus been interpreted as a tragedy of the commons, since developers and users of antibiotics do not need to take into account the negative (long-term) consequences of increased resistance (Hollis and Maybarduk 2015). Hence, as long as this externality is not addressed, we can assume that the share of resistant bacteria is excessive and may further be affected by the pricing policies of pharmaceutical companies (Herrmann 2010).

Second, even if excessive use of antibiotics is avoided, without the development of new antibiotics, the share of resistant bacteria tends to rise and will continuously reemerge, even to new compounds (McKenna 2020). This generates large welfare losses due to sickness and premature deaths. In turn, according to a report of the European Court of Auditors (2019), “the antimicrobials market lacks commercial incentives to develop new treatments”. In particular, the development costs of new antibiotics can amount to more than one billion USD and the probability of a successful development might be only a few percent (Payne et al. 2007; DiMasi et al. 2016; Årdal 2020). Together with the targeted and limited use of new antibiotics (i.e., low initial sales volumes), the development of new antibiotics is regarded as a very risky business model compared to other development options. As a consequence, under current market conditions, investments in the development of new antibiotics—and in particular narrow-spectrum antibiotics targeted against resistant bacteria–are not commercially attractive (McKenna 2020). Thus, stimulating such developments is a second—and probably the most important—task for policy.

The objective of policy is thus to devise strategies that help incentivizing pharmaceutical companies to focus on the development of narrow-spectrum antibiotics. In the aforementioned report of the European Court of Auditors (2019), it is suggested that some of the EU AMR research budget should be reallocated to generate new economic incentives for pharmaceutical companies (Watson 2019). In the USA, the Generating Antibiotic Incentives Now (GAIN) Act from 2012 pursues similar goals by “stimulating the development and approval of new antibacterial and antifungal drugs” (FDA 2017).

*A Refunding Approach*. In this paper, we develop a complementary approach by constructing a refunding scheme for successful developments of antibiotics, which does not rely on the use of taxpayer money. In particular, we propose a dynamical refunding mechanism that rewards companies that have successfully developed a new antibiotic. It works as follows. A successful company can claim a refund from an antibiotics fund to partially cover its development costs. The proposed refund involves a fixed and a variable part. The variable part increases with the use of the new antibiotic for *currently resistant* strains in comparison with other newly developed antibiotics for the same purpose—“the resistance premium”—and decreases with the use of this antibiotic for non-resistant bacteria—“the non-resistance penalty.” With an appropriate choice of refunding parameters, it becomes commercially attractive to develop a narrow-spectrum antibiotic, or to switch to such an antibiotic if the development becomes feasible in the R &D process. Developing new broad-spectrum antibiotics should be less attractive, but if they can be used against resistant bacteria, this should also be commercially viable. The antibiotics fund, in turn, is continuously financed by fees levied on the non-human use of *existing* antibiotics and should be started by initial contributions from the industry and public institutions like the recently established AMR Action fund.^2^

**Fig. 1:**
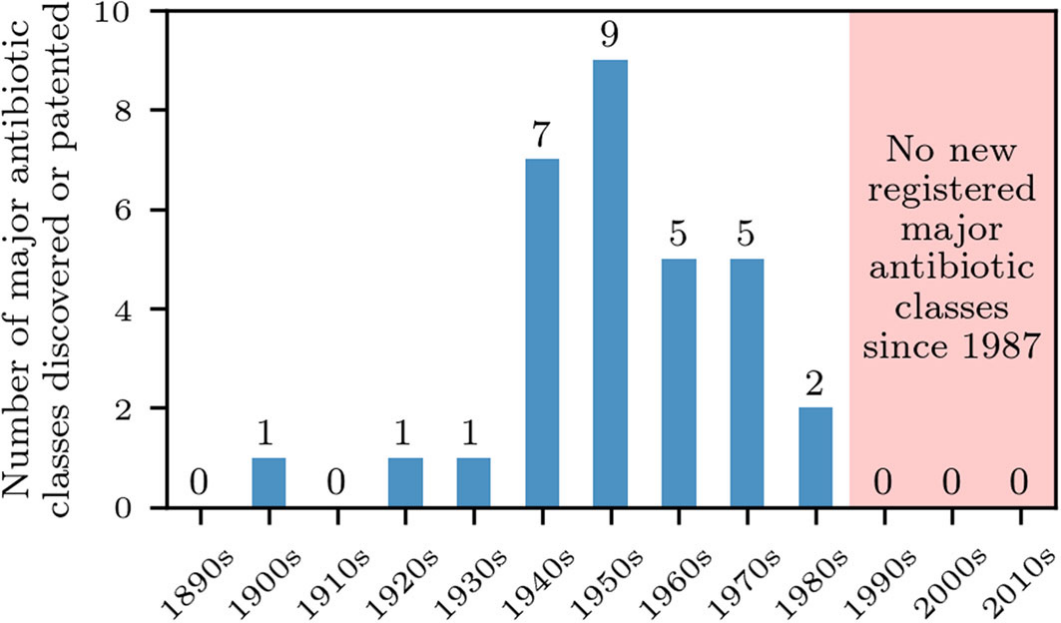
(Color figure online) The discovery void. We show the number of registered or patented major antibiotic classes from 1890–2020 (Silver 2011; Talkington et al. 2016). Although antibiotics have been registered after 1987 (e.g., plazomicin in 2018 (FDA 2018a) and lefamulin in 2019 (FDA 2019)), the majority of the corresponding chemical classes was registered (or first isolated) many years before [e.g., aminoglycosides in 1944 (Krause et al. 2016) and pleuromutilins in 1952 (Silver 2011)]

*Broader Perspective*. It is useful to place our proposal into a broader context. First, incentivizing the development of narrow-spectrum antibiotics has to be matched by the development and use of efficient diagnostic techniques to quickly and precisely determine the type of bacterial strain that causes a health problem and by collecting information regarding the type of bacterial strain, the optimal treatment, and its outcome. Many OECD countries have already implemented extended reporting systems (see, e.g., the Swiss antibiotic strategy 2015).

In addition to the development of narrow-spectrum agents, it is important to consider alternative approaches such as medication that sustains and boosts the human immune system during infections, or improved sterilization and sanitation in hospitals (Maxson and Mitchell 2016). Other strategies for fighting bacterial infections, such as targeting virulence and treatments with antibodies or phage (Kutateladze and Adamia 2008; Gordillo Altamirano and Barr 2019; Kortright et al. 2019), are alternatives to antibiotics.

In practice, it will not be easy to encourage pharmaceutical companies to refocus their R &D activities. The disappointing finding that genomics did not lead to many new classes of antibiotics caused the close-down of many antibiotic research laboratories (Coates et al. 2011; Laxminarayan 2013). In the past 30 years, antibiotic R &D efforts were rather limited (see Fig. 1) because of the huge development costs and low expected returns. Martin et al. (2017) analyzed the clinical trial costs of 726 studies that were conducted between 2010 and 2015. In the initial clinical trial phase, the median cost was found to be 3.4 million USD and the median cost of phase III clinical trials^3^ was reported to be more than 20 million USD. High development costs of antibiotic drugs limit the number of players in this area and require major companies to be involved in the development process. A good research ecosystem for antibiotic development necessarily involves large companies, entailing significant in-house efforts, but also collaborations with academia, buying or investing in SMEs, and joint ventures with other large pharmaceutical companies. An appropriately designed refunding scheme can help to foster such an R &D ecosystem, as we discuss in Sect. 4.

A recent report published by the European Observatory on Health Systems and Policies (Anderson et al. 2019) suggests a multifold R &D approach to combat AMR. It includes: (i) push incentives (e.g., direct funding and tax incentives) and pull incentives (e.g., milestone prize and patent buyout) for the development of new antibiotics, (ii) research in diagnostics (e.g., rapid tests to distinguish between bacterial and viral infections), and (iii) vaccine research. Our proposed refunding scheme involves both strong push and pull incentives to foster the development of new antibiotics.

Finally, to fix the broken antibiotic market, the National Health Service (NHS) of the UK established a subscription-based payment model in 2019 (NHS 2019). The strategy of the NHS is to pay pharmaceutical companies a fixed amount for using their antibiotics, based on the benefits of the antibiotic for society. In this way, the return is not proportional to sales volumes anymore. This, in turn, may help to limit the emergence of antibiotic resistance, since the underlying a market-entry reward makes the development of new antibiotics financially more attractive, even if the use of the antibiotic is not widespread.

Our refunding approach can contribute to such strategies in three ways: First, the refunding scheme provides a specific approach to implement which antibiotic use is beneficial for a society, namely by introducing rewards such as the “resistance premium” and reductions in these rewards when antibiotics are used for non-resistant bacteria. Otherwise, it may not be clear what the benefit for society is when a new antibiotic is developed, since the benefit depends on its use later on. Second, the refunding approach is applicable in standard market scenarios where the return is still determined by sales volumes. Third, it can work without using taxpayer funds if the antibiotic fund is filled by a levy on antibiotic use.

*Organization of the Paper*. Based on a general antibiotic resistance modeling framework, which we derive in the Appendix, we formulate a model variant in Sect. 2 that allows us to study the antibiotics dilemma: Developing narrow-spectrum antibiotics, which are only effective against specific bacterial strains, is most beneficial for society, but least attractive for pharmaceutical companies, due to their limited usage and sales volumes. We couple this variant of the general antibiotics model to our refunding scheme in Sect. 3 and illustrate how refunding can lead to better treatment protocols and a lower share of resistant strains. In Sect. 4, we outline possibilities to design refunding schemes for antibiotic resistance dynamics with more than two antibiotics and various forms of R &D uncertainties and discuss how refunding schemes can promote the biotech ecosystem. We discuss and conclude our study in Sect. 5.

## Narrow-Spectrum Versus Broad-Spectrum Antibiotics

### Research and Development Opportunities

To provide a formal representation of both the antibiotics dilemma and the refunding scheme, we first focus on resistance dynamics and refunding schemes for $n=2$ antibiotics. In Sect. 4, we discuss a more general refunding approach that builds on a general antibiotic resistance model, which we derive in Appendix A.

For $n=2$ antibiotics ($\mathrm A$ and $\mathrm B$), there are $N=2^2=4$ infected compartments associated with different degrees of antibiotic resistance. The sets of antibiotics are ${\mathcal {A}}_1=\{\mathrm {A},\mathrm {B}\}$ (wild type, susceptible to treatment with antibiotics $\mathrm {A}$ and $\mathrm {B}$), ${\mathcal {A}}_2=\{\mathrm {A}\}$ (susceptible to treatment with antibiotic $\mathrm {A}$), ${\mathcal {A}}_3=\{\mathrm {B}\}$ (susceptible to treatment with antibiotic $\mathrm {B}$), and ${\mathcal {A}}_4=\emptyset$ (completely resistant).

We assume that antibiotic $\mathrm A$ is already on the market. For the development of a second antibiotic $\mathrm B$, pharmaceutical companies have two different options.

(i) Antibiotic $\mathrm {B}_1$: This is a broad-spectrum antibiotic that is as effective as antibiotic $\mathrm {A}$ against wild-type strains. It is also effective against strains that are resistant against $\mathrm {A}$.
(ii) Antibiotic $\mathrm {B}_2$: This is a narrow-spectrum antibiotic that is, by a factor $1-\epsilon \in [0,1]$, less effective against wild-type strains. However, antibiotic $B_2$ is, by a factor $1+\epsilon$, more effective against strains that are resistant to $\mathrm {A}$ than $\mathrm {A}$ is effective against strains that are resistant to antibiotic $\mathrm {B}_2$.

The higher effectiveness of antibiotic $\mathrm {B}_2$ against $\mathrm {A}$-resistant strains makes this drug preferable over antibiotic $\mathrm {B}_1$ for treating infections that result from those bacterial strains. The described differences in effectiveness between narrow- and broad-spectrum antibiotics have been observed clinically (Palmer et al. 1995). In the remainder of this manuscript, we classify antibiotics according to their effectiveness against certain bacterial strains into the categories “narrow” and “broad.”^4^

We will later turn to costs and chances associated with the development of antibiotics $\mathrm {B}_1$ and $\mathrm {B}_2$, but first study their effect on the evolution of resistance.

### The Model

**Fig. 2:**
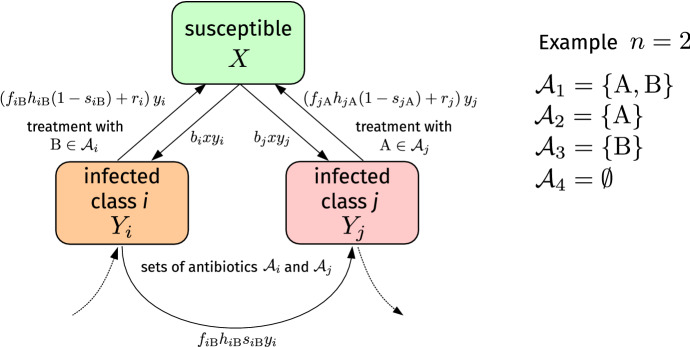
(Color figure online) Model schematic. Susceptible individuals (i.e., individuals in state *X*) can be infected by individuals in state $Y_i$ (i.e., individuals who are infected with bacterial strain *i*) at rate $b_i$. Infected individuals in state $Y_i$ recover spontaneously at rate $r_i$. The effective antibiotic-induced recovery rate associated with individuals in state $Y_i$ and a certain antibiotic $\mathrm {B}$ is $f_{i\mathrm {B}} h_{i\mathrm {B}}$. Only a fraction $1-s_{i\mathrm {B}}$ of individuals in state $Y_i$ recovers after a treatment with antibiotic $\mathrm {B}$. The remaining fraction $s_{i\mathrm {B}}$ becomes resistant against antibiotic $\mathrm {B}$ and ends up in a compartment $Y_j$ of bacterial strains exhibiting more resistances. The sets of effective antibiotics in compartments $Y_i$ and $Y_j$ are ${\mathcal {A}}_i$ and ${\mathcal {A}}_j$, respectively. Infection and recovery processes with the respective rates are also present in compartment $Y_j$. For $n=2$ antibiotics, we show the possible antibiotic-treatment classes ${\mathcal {A}}_1,{\mathcal {A}}_2,{\mathcal {A}}_3,{\mathcal {A}}_4$. We account for birth and death dynamics in the antibiotic resistance model defined by Eq. (1) (not shown in the model schematic for the sake of brevity)

In this section, we formulate the population-level resistance dynamics for both types of antibiotics $\mathrm {B}_1$ and $\mathrm {B}_2$. A detailed summary of an antibiotic resistance model with *n* antibiotics is provided in Appendix A.

We describe the evolution of resistances in a population in terms of a susceptible-infected-susceptible-type (SIS-type) model (Keeling and Rohani 2011; Bonhoeffer et al. 1997; Levin and Bonten 2004; Uecker and Bonhoeffer 2021) (see Fig. 2). We use *X* and $Y_i$ ($1\le i\le 4$) to denote susceptible and infected states, respectively. Individuals in state $Y_i$ can be treated with antibiotics that are elements of the set ${\mathcal {A}}_i$ (see Sect. 2.1). The corresponding rate equations are

1 $$\begin{aligned} \frac{\mathrm {d} x}{\mathrm {d} t}&=-b x \left( y_1+y_2+y_3+y_4\right) +r_1 y_1+r_2 y_2 + r_3 y_3+r_4 y_4 \nonumber \\&+ h (1-s) \left( y_1\left[ f_{1\mathrm {A}}+(1-\epsilon )f_{1\mathrm {B}}\right] +y_2 +y_3(1+\epsilon )\right) +\lambda - d x,\nonumber \\ \frac{\mathrm {d} y_1}{\mathrm {d} t}&=\left[ b x - r_1 -c-h\left( f_{1\mathrm {A}}+(1-\epsilon ) f_{1\mathrm {B}}\right) \right] y_1, \nonumber \\ \frac{\mathrm {d} y_2}{\mathrm {d} t}&=\left( b x - r_2 -c-h\right) y_2+ h s (1-\epsilon ) f_{1\mathrm {B}} y_1, \nonumber \\ \frac{\mathrm {d} y_3}{\mathrm {d} t}&=\left( b x - r_3-c-h (1+\epsilon ) \right) y_3+ h s f_{1 \mathrm {A}} y_1, \nonumber \\ \frac{\mathrm {d} y_4}{\mathrm {d} t}&=\left( b x - r_4-c\right) y_4 + h s\left[ y_2+(1+\epsilon )y_3 \right] . \end{aligned}$$

Here, *x* denotes the proportion of susceptible individuals and $y_i$ ($1\le i\le 4$) is the proportion of individuals infected by strains with different degrees of resistance ($i=1$: wild-type, $i=2$: susceptible to treatment with antibiotic $\mathrm A$, $i=3$: susceptible to treatment with antibiotic $\mathrm B$, $i=4$: completely resistant).^5^ In Eq. (1), we use a constant infection rate and a constant fraction of individuals that develop antibiotic resistance, i.e. $b_i=b$ and $s_{ij}=s$. The birth rate of new susceptible individuals is $\lambda$ and the corresponding death rate is *d*. For infected individuals, the death rate is *c*. The antibiotic-induced recovery rates are $h_{1\mathrm {A}}=h$, $h_{1\mathrm {B}}=h(1-\epsilon )$, $h_{2 \mathrm {A}}=h$, and $h_{3 \mathrm {B}}=h (1+\epsilon )$. Other parameter choices do not affect the mathematical structure of the dynamical system (1), which we analyze in the subsequent sections. The assumption of an equal infection rate *b* of different strains is justified by corresponding empirical findings (Chehrazi et al. 2019). For modeling antibiotic $\mathrm {B}_1$, we simply set $\epsilon =0$. If antibiotic $\mathrm {B}_2$ is present, we set the values of these parameters to the effectiveness disadvantages and advantages of $\mathrm {B}_2$ relative to $\mathrm {A}$.

In the main text, we set the recovery rate $h=1$ and we assume a small value of the proportion of individuals who develop resistant strains ($s= 0.05$). The effective time scale of recovery (associated with the rate $(1-s)h$) may thus appear much shorter than that of the emergence of resistance (associated with the rate *sh*). If one wishes to adjust the distribution of the probability of having acquired resistance to a specific antibiotic at a certain time after the initiation of therapy, one can explicitly account for the time since the start of antibiotic therapy and formulate an age-structured version (i.e., Kermack–McKendrick-type models as described by M’Kendrick 1925; Chou and Greenman 2016) of antibiotic resistance dynamics.

We now focus on four different treatment protocols:

I. Treatment with antibiotic $\mathrm {B}_1$ and symmetric use of antibiotics (i.e., half of the patients in state $Y_1$ receive antibiotic $\mathrm A$ ($\mathrm{B}_1$), a “50/50” treatment protocol):
  Treatment with antibiotic $\mathrm {B}_1$ implies that $\epsilon =0$. Moreover, since we consider a symmetric use, $f_{1 \mathrm {A}}=0.5$ and $f_{1 \mathrm {B}}=0.5$, in this treatment protocol 50% of patients with a wild-type-strain infection receive antibiotic $\mathrm {A}$ and the remaining 50% receive antibiotic $\mathrm {B}_1$. Both antibiotics $\mathrm {A}$ and $\mathrm {B}_1$ have the same effect on strains 1 and 2 and 1 and 3, respectively.
II. Treatment with antibiotic $\mathrm {B}_1$ and asymmetric use of antibiotics (i.e., a “100/0” treatment protocol):
  We again have $\epsilon =0$ and we use the new antibiotic $\mathrm {B}_1$ only against strains that are resistant against $\mathrm {A}$. All patients with a wild-type-strain infection receive antibiotic $\mathrm {A}$, i.e. $f_{1 \mathrm {A}}=1$ and $f_{1 \mathrm {B}}=0$.
III. Treatment with antibiotic $\mathrm {B}_2$ and symmetric use of antibiotics:
  The symmetric use implies $f_{1 \mathrm {A}}/f_{1 \mathrm {B}}=50/50$ for wild-type-strain infections. The prefactor $1-\epsilon$ accounts for the corresponding recovery-rate difference in the wild-type compartment. However, antibiotic $\mathrm {B}_2$ is more effective in the infected compartment 3, where individuals have an antibiotic-induced recovery rate of $h (1+\epsilon )$.
IV. Treatment with antibiotic $\mathrm {B}_2$ and asymmetric use of antibiotics:
  Here, we only use antibiotic $\mathrm {A}$ in the first infected compartment and we set $f_{1 \mathrm {A}} = 1$ and $f_{1 \mathrm {B}}=0$.

### Performance Measures

The performance of the proposed treatment protocols can be compared in terms of different measures including the total stationary population

2 $$\begin{aligned} P^*:=x^*+\sum _{i=1}^N y_i^*, \end{aligned}$$

where the asterisk denotes the stationary densities of *x* and $y_i$. Another possible performance measure is the gain of healthy individuals

3 $$\begin{aligned} G(T):=\int _0^{T} x(t)\, \mathrm {d}t-\int _0^{T}x(t;h_{ij}=0) \mathrm {d}t, \end{aligned}$$

resulting from antibiotic treatment during some time, denoted by *T*, where $x(t;h_{ij}=0)$ denotes the proportion of susceptible individuals in the absence of treatment (i.e., $h_{ij}=0$ for all *i*, *j*).

**Fig. 3:**
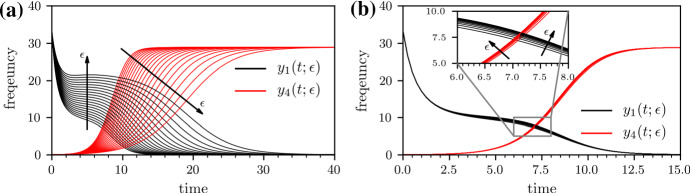
(Color figure online) Growth of multi-resistant strains under 50/50 and 100/0 treatment. The evolution of the proportion of individuals infected by wild-type strains, $y_1$, (solid black line) and completely resistant strains, $y_4$, (solid red line) under (**a**) 50/50 treatment with $f_{1\mathrm {A}}=f_{1\mathrm {B}_2}=0.5$ (**b**) and 100/0 treatment with $f_{1\mathrm {A}}=1$ and $f_{1\mathrm {B}_2}=0$. To obtain the shown solutions, we numerically solve Eq. (1) with a classical Runge–Kutta scheme in the time interval [0, *T*] with $T=100$ for $\lambda =100$, $d=1$, $c=1.5$, $b=0.03$, $r_{i}=(2-k_i) 0.1$ ($k_i$ is the number of effective antibiotics in the *i*th infected compartment), $h=1$, $s=0.05$, and $\epsilon \ge 0$. The initial conditions are $x(0)=50$, $y_1(0)=33.33$, $y_2(0)=y_3(0)=y_4(0)=0$

Finally, we may wish to calculate the time at which half of the infected individuals are infected by bacterial strains that are resistant against any antibiotic. This “half-life” of non-resistance is

4 $$\begin{aligned} T_{1/2}:=\left\{ t\bigg |\frac{y_N(t)}{\sum _{i=1}^{N} y_i(t)}=\frac{1}{2}\right\} . \end{aligned}$$

As described in Appendix B and as proved in Appendix C, the long-term stationary population $P^*$ is not a suitable performance measure, because $P^*$ is identical for all treatment protocols that we will consider in the following sections. However, both *G*(*T*) and $T_{1/2}$ are suitable measures to compare different development strategies of antibiotics. In addition, we use $G_{1/2}:=G(T_{1/2})$ as a complementary performance measure that quantifies the gain of healthy individuals at half-life of non-resistance.

### Comparisons

**Fig. 4:**
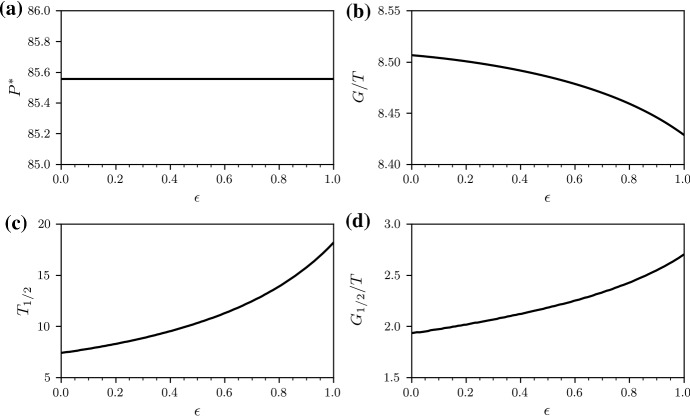
Treatment with two antibiotics (50/50 strategy). Performance measures (**a**) $P^*$, (**b**) *G*(*T*)/*T*, (**c**) $T_{1/2}$, and (**d**) $G_{1/2}/T$ with $G_{1/2}:=G(T_{1/2})$ under treatment with two antibiotics (50/50 strategy). We numerically solve Eq. (1) with a classical Runge–Kutta scheme in the time interval [0, *T*] with $T=100$ for $\lambda =100$, $d=1$, $c=1.5$, $b=0.03$, $r_{i}=(2-k_i) 0.1$ ($k_i$ is the number of effective antibiotics in the *i*th infected compartment), $h=1$, $s=0.05$, $f_{1\mathrm {A}}=f_{1\mathrm {B}_2}=0.5$, and $\epsilon \ge 0$. The initial conditions are $x(0)=50$, $y_1(0)=33.33$, $y_2(0)=y_3(0)=y_4(0)=0$

**Fig. 5:**
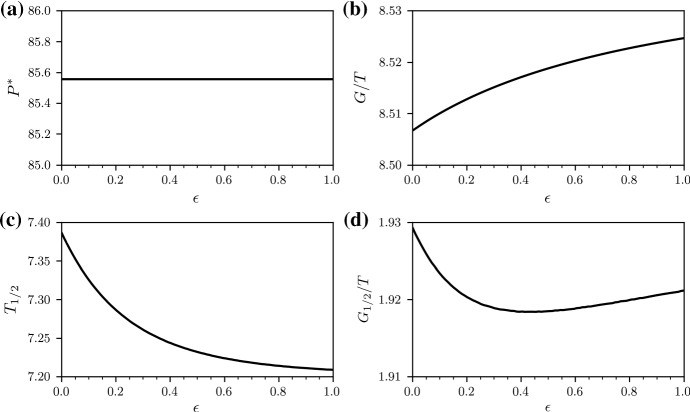
Treatment with two antibiotics (100/0 strategy). Performance measures (**a**) $P^*$, (**b**) *G*(*T*)/*T*, (**c**) $T_{1/2}$, and (**d**) $G_{1/2}/T$ with $G_{1/2}:=G(T_{1/2})$ under treatment with two antibiotics (100/0 strategy). We numerically solve Eq. (1) with a classical Runge–Kutta scheme in the time interval [0, *T*] with $T=100$ for $\lambda =100$, $d=1$, $c=1.5$, $b=0.03$, $r_{i}=(2-k_i) 0.1$ ($k_i$ is the number of effective antibiotics in the *i*th infected compartment), $h=1$, $s=0.05$, $f_{1\mathrm {A}}=1$, $f_{1\mathrm {B}_2}=0$, and $\epsilon \ge 0$. The initial conditions are $x(0)=50$, $y_1(0)=33.33$, $y_2(0)=y_3(0)=y_4(0)=0$

**Fig. 6:**
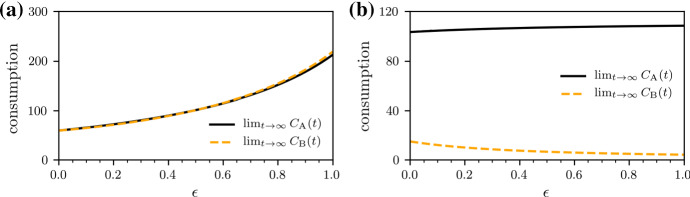
(Color figure online) Antibiotic consumption. The stationary antibiotic consumption $\lim _{t \rightarrow \infty }C_{\mathrm{A}}(t)$ and $\lim _{t \rightarrow \infty } C_{\mathrm{B}}(t)$ for (**a**) 50/50 treatment with $f_{1\mathrm {A}}=f_{1\mathrm {B}_2}=0.5$ and (**b**) 100/0 treatment with $f_{1\mathrm {A}}=1$ and $f_{1\mathrm {B}_2}=0$. We numerically solve Eq. (1) with a classical Runge–Kutta scheme in the time interval [0, *T*] with $T=100$ for $\lambda =100$, $d=1$, $c=1.5$, $b=0.03$, $r_{i}=(2-k_i) 0.1$ ($k_i$ is the number of effective antibiotics in the *i*th infected compartment), $h=1$, $s=0.05$, and $\epsilon \ge 0$. The initial conditions are $x(0)=50$, $y_1(0)=33.33$, $y_2(0)=y_3(0)=y_4(0)=0$

We now compare treatment protocols I–IV for $\epsilon \in [0,1]$ in terms of the total stationary population, $P^*$, gain of healthy individuals, *G*, half-life of non-resistance, $T_{1/2}$, and the half-life gain $G_{1/2}:=G(T_{1/2})$.

We first study differences between the evolution of the wild-type and fully resistant compartments under 50/50 and 100/0 treatment. Figure 3 shows that the variability in $y_1$ and $y_4$ is much larger under 50/50 treatment than under 100/0 treatment. Comparing the performance measures $P^*$, *G*, $T_{1/2}$, and $G_{1/2}$ (see Figs. 4 and 5), we find that the 50/50 and 100/0 treatments are equivalent for $\epsilon =0$. For larger values of $\epsilon$, the gain *G* of the 50/50 treatment is smaller than the gain of the 100/0 treatment.

We complement the above comparison of 50/50 and 100/0 treatments by monitoring the use of antibiotics $\mathrm A$, $\mathrm {B}_1$, and $\mathrm {B}_2$, respectively. We can keep track of the consumption of antibiotics $\mathrm A$ and $\mathrm B$ by integrating

5 $$\begin{aligned} \frac{\mathrm {d} C_{\mathrm{A}}}{\mathrm {d} t} = f_{1 \mathrm A} y_1 + y_2\quad \text {and} \quad \frac{\mathrm {d} C_{\mathrm{B}}}{\mathrm {d} t} = f_{1 \mathrm B} y_1 + y_3 \end{aligned}$$

over time. Differences between the two treatments are also reflected in the final consumption $C_{\mathrm{A}}$ and $C_{\mathrm{B}}$ of antibiotics $\mathrm A$ and $\mathrm B$ (see Fig. 6). The 100/0 treatment protocol is associated with the minimum use of the valuable antibiotic $\mathrm{B}_2$, and it thus leads to the largest refund as defined in the next section. For $\epsilon \gtrsim 0.5$, the 100/0 treatment leads to a significantly lower consumption of both antibiotics, compared to the 50/50 protocol.

Our analysis highlights a fundamental dilemma. Developing a narrow-spectrum antibiotic $\mathrm {B}_2$ is highly beneficial for society, but this antibiotic should only be used very little, namely against the strains which are resistant against antibiotic $\mathrm A$. We refer to this issue as the *antibiotics dilemma*: Developing a narrow-spectrum antibiotic against resistant bacteria is most attractive for society, but least attractive for companies, since usage should be limited, so that sales are low.

The situation is further complicated by additional properties of antibiotics development and usage. First, the development costs are enormous, in the range of about one billion USD, and second, the chances to succeed are low. This is true in general for new drugs (DiMasi et al. 2016) but more pronounced for antibiotics, where the success probability may be as low as 5% (Årdal 2020).

## Refunding Schemes

### The Basic Principles

To overcome the antibiotics dilemma and associated complications, we propose a refunding scheme to incentivize the development and appropriate use of antibiotics. The main properties of the refunding scheme are as follows:

1. An antibiotics fund should be started with initial contributions from industry and public institutions, similar to the recently established AMR Action fund. In addition, all antibiotic use is charged with a small fee which is channeled continuously into the antibiotics fund.
2. Firms that develop new antibiotics obtain a refund from the fund.
3. The refund for a particular antibiotic is calculated with a formula that satisfies the following three properties:
  There is a fixed payment for the successful development of an antibiotic, i.e., an antibiotic that is approved by the public health agency responsible for such approvals (e.g., the U.S. Food and Drug Administration (FDA)). This part is in the spirit of Kremer (1998), as it is equivalent to an advanced market commitment. Pharmaceutical companies know that once a patent for a new antibiotic is awarded, they will be reimbursed part of their development costs.
  The refund is strongly increasing with the use of the new antibiotic for currently resistant bacteria, compared to other newly developed antibiotics for this purpose. This part is the *resistance premium*.
  The refund is declining in the use of the antibiotics for non-resistant bacteria, compared to other antibiotics used for this purpose. This part is the *non-resistance penalty*.

The objective of our refunding scheme is to financially incentivize pharmaceutical companies to reorient their R &D efforts and sales strategy toward narrow-spectrum antibiotics, using a minimum-size antibiotics fund. As we will demonstrate below, all above elements (1–3) are necessary to achieve this purpose.

Several remarks are in order to summarize the application areas of refunding schemes and the challenges arising in the context of antibiotic resistance. First, refunding schemes are widely discussed in the environmental literature. These schemes are meant to provide incentives for firms to reduce pollution (Gersbach and Winkler 2012). Second, simple forms of refunding schemes could also be used in other contexts where pharmaceutical companies have only little financial interest in investing in drug research, due to potentially low sales volumes. This is, for instance, the case for orphan drug development and vaccine research for viral infections, including SARS and Ebola, or enduring epidemic diseases as described by Bell and Gersbach (2009). However, for such cases, refunding schemes are much easier to construct, since they can solely rely on the usage, e.g. the number of vaccinated individuals. For antibiotics—because of the antibiotics dilemma—one has to construct new types of refunding schemes with “sticks and carrots”: The carrots for using the antibiotic against bacterial strains resistant against other antibiotics and the sticks for using the antibiotics against wild-type strains. This stick-and-carrot complication does not arise in the context of the aforementioned (simple) refunding schemes.

### Refunding Schemes for Two Antibiotics

We illustrate the working of the refunding scheme with a simple model. It includes two elements:

- There is a fixed amount, denoted by $\alpha$, which a pharmaceutical company obtains if it successfully develops a new antibiotic $\mathrm {B}_i$, i.e. an antibiotic approved by a public health authority.
- There is a variable refund that is determined by the following refunding function:
  6 $$\begin{aligned} g(f_{1 \mathrm {B}_i}y_1, f_{3\mathrm {B}_i}y_3) = \beta \frac{f_{3\mathrm {B}_i}y_3}{\gamma f_{1\mathrm {B}_i}y_1+f_{3\mathrm {B}_i}y_3}, \end{aligned}$$
  where $i\in \{1,2\}$ (to represent antibiotics $\mathrm {B}_1$ and $\mathrm {B}_2$) and $\beta$ and $\gamma$ are scaling parameters, with $\beta$ being a large number and $\gamma$ satisfying $\gamma \ge 0$. The parameter $\beta$ determines the refund per drug unit and $\gamma$ controls the non-resistance penalty. The refunding function $g(f_{1\mathrm {B}_i}y_1, f_{3\mathrm {B}_i}y_3)$ measures the relative use of the new antibiotic in compartment 3 (A-resistant strains) compared to the total use of the antibiotic. The use of the antibiotic in the wild-type compartment is weighted by the parameter $\gamma$. Note that the refunding function *g* satisfies the following properties.
  - It is bounded according to $0 \le g(f_{1\mathrm {B}_i}y_1, f_{3\mathrm {B}_i}y_3) \le \beta$.
  - It is increasing in the use for $\mathrm A$-resistant bacteria in comparison with other newly developed antibiotics used for this purpose: $f_{3\mathrm {B}_i}y_3$.
  - It is declining in the use of antibiotics for non-resistant bacteria in comparison with other antibiotics used for this purpose: $f_{1\mathrm {B}_i}y_1$.
  - It reaches a maximum if the antibiotic is only used to treat A-resistant strains and 0 if it is only used for non-resistant strain treatment.

Note that our refunding scheme uses three free parameters $\alpha$, $\beta$, and $\gamma$. We will show in the subsequent section that all three parameters are necessary to achieve the objective of the refunding scheme.

The total refund that a successful pharmaceutical company receives in the time interval [0, *T*] for developing an antibiotic $\mathrm {B}_i$ is given by

7 $$\begin{aligned} R_i(T) :=\alpha +\int _0^{T} \beta \frac{f_{3\mathrm {B}_i}y_3 (f_{1\mathrm {B}_i}y_1+f_{3\mathrm {B}_i}y_3)}{\gamma f_{1\mathrm {B}_i}y_1+f_{3\mathrm {B}_i}y_3}\, \mathrm {d}t. \end{aligned}$$

For $\gamma =1$, the refund is solely determined by $f_{3 \mathrm {B}_i}y_3$ and the use of antibiotics in compartment 1 is irrelevant for the refund. For $\gamma >1$, the use of antibiotics in compartment 1 decreases the refund, and thus the use of the antibiotics for non-resistant bacteria is penalized. As we will see in our numerical examples, for small values of $\epsilon$ (see Eq. (1)), such penalties may not always be needed, but we certainly need them for higher values of $\epsilon$.

### Incentivizing Development

**Table 1:** Overview of the main refunding scheme parameters. The values that are listed in the last column are used to perform a calibration of the refunding scheme in Sect. 3.5. We use the same parameters for antibiotics $\mathrm {B}_1$ and $\mathrm {B}_2$ (i.e., for $i=1,2$)

Quantity	Symbol	Value
Success probability	$q_i$	0.1
Development costs	$K_i$	2 billion USD
Revenue per unit	$p_i$	100 USD
Production costs per unit	$v_i$	70 USD
Refunding offset	$\alpha$	1 billion USD
Refund per unit	$\beta$	to be determined (see Sect. 3.5)
Non-resistance penalty	$\gamma$	to be determined (see Sect. 3.5)

We next focus on how our refunding scheme can incentivize a pharmaceutical company to invest in R &D for new antibiotics and in particular for new narrow-spectrum antibiotics. We assume that the pharmaceutical company makes a risk-neutral evaluation of such R &D investments.^6^ For this purpose, we first consider the situation without refunding. For simplicity, we neglect discounting. Then, without refunding (i.e., without $R_i(T)$), the net profit of the company under consideration that invests into the development of an antibiotic $\mathrm {B}_i$ is

8 $$\begin{aligned} \pi _i = q_i (p_i-v_i) \int _0^T \frac{\mathrm {d} C_{\mathrm {B}_i}}{\mathrm {d}t}\, \mathrm {d}t - K_i = q_i (p_i-v_i) \int _0^{T} (f_{1 \mathrm {B}_i}y_1+f_{3\mathrm {B}_i}y_3)\, \mathrm {d}t - K_i,\nonumber \\ \end{aligned}$$

where $K_i$ denotes the total development costs of $\mathrm {B}_i$, and $q_i$ is the probability of success when the development is undertaken. Moreover, $p_i$ is the revenue per unit of the antibiotic used in medical treatments for the pharmaceutical company under consideration, and $v_i$ are the production costs per unit. An overview of the main parameters used in our refunding scheme is provided in Table 1.

Note that in our example with two antibiotics, $f_{3 \mathrm {B}_i}=1$, since only drug $\mathrm {B}_i$ can be used against A-resistant strains. We assume that without refunding, $\pi _i$ is (strongly) negative, because of high development costs $K_i$ and low success probabilities $q_i$. The task of a refunding scheme is three-fold: First, it has to render the development of new antibiotics commercially viable. Second, it has to render the development of narrow-spectrum antibiotics against resistant bacteria strains more attractive than the development of broad-spectrum antibiotics. Third, if a narrow-spectrum antibiotic is developed that is also effective against wild-type strains, but less so than others, the refunding scheme should make its use against wild-type strains unattractive.

With a refunding scheme in place, we directly look at the conditions for such a scheme to achieve the break-even condition, i.e., a situation at which $\pi _i$ becomes zero and investing into antibiotics development just becomes commercially viable. We assume that the pharmaceutical company continues to receive $p_i$ per unit of the antibiotic sold.^7^

The general break-even condition for a newly developed antibiotic $\mathrm {B}_i$ is

9 $$\begin{aligned} \begin{aligned} K_i&= q_i (p_i-v_i) \int _0^{T} (f_{1\mathrm {B}_i}y_1+f_{3\mathrm {B}_i}y_3) \, \mathrm {d}t + q_i R_i(T)\\&= \alpha q_i + q_i\int _0^{T} \left[ \beta \frac{f_{3\mathrm {B}_i}y_3}{\gamma f_{1\mathrm {B}_i}y_1+f_{3\mathrm {B}_i}y_3} + (p_i-v_i)\right] (f_{1\mathrm {B}_i}y_1+f_{3\mathrm {B}_i}y_3) \, \mathrm {d}t. \end{aligned} \end{aligned}$$

Clearly, refunding increases the profits from developing new antibiotics since $R_i(T)>0$. There are many combinations of the refunding parameters $\alpha$, $\beta$, and $\gamma$ that can achieve this break-even condition. However, and more subtly, the refunding has to increase the incentives for the development of narrow-spectrum antibiotics more than those for broad-spectrum antibiotics. This can be achieved by an appropriate choice of the scaling parameter, as we will illustrate next.

In Eq. (9), we have (implicitly) assumed that there is a life-time *T* for the drug and that the company wants to achieve break-even over that period. There are two caveats to this assumption.

First, some (smaller) biotech companies cannot raise enough capital in the market to finance the initial development, as financiers prefer immediate over future rewards. Hence, such companies would need to achieve profits above break-even levels in order to be attractive for investors, as the investment is long term.

Second, we have neglected many sources of uncertainty about the future revenues the new antibiotic will generate, such as uncertainties about prices, volume, life time (including new antibiotics produced by competitors), and production costs. Such uncertainties will typically call for additional risk premia that have to be added to the break-even condition. Or, in other words, the break-even condition in expected terms has to be achieved in a shorter time period. Typically, such time periods can be in the range of five to ten years or a bit more, but not much longer.

### Critical Conditions for Refunding Parameters

To derive the critical refunding parameters, we assume that the parameter $\alpha$, which satisfies $0< \alpha < K_i$, is given, and thus, a fixed share of the R &D costs is covered by the antibiotics fund. We also assume that $\alpha +\pi _i<K_i$, where $\pi _i$ is the profit without refunding. Based on the break-even condition (see Eq. (9)), we obtain the following general condition that the parameters $\beta$ and $\gamma$ have to satisfy:

10 $$\begin{aligned} \beta = \frac{K_i-\alpha q_i-q_i(p_i-v_i)\int _0^T(f_{1 \mathrm {B}_i}y_1 + f_{3 \mathrm {B}_i}y_3)\mathrm {d}t}{q_i\int _0^T \frac{f_{3 \mathrm {B}_i}y_3}{\gamma f_{1 \mathrm {B}_i}y_1 + f_{3 \mathrm {B}_i}y_3}(f_{1 \mathrm {B}_i}y_1+f_{3 \mathrm {B}_i}y_3)\mathrm {d}t}. \end{aligned}$$

The goal of our refunding scheme is to incentivize pharmaceutical companies to produce narrow-spectrum antibiotics $\mathrm {B}_2$ that are only used against currently resistant strains (see treatment IV in Sect. 2). Thus, the refunding scheme has to satisfy two conditions: first, with the development of antibiotic $\mathrm {B}_2$, the company achieves break-even. Second, developing antibiotic $\mathrm {B}_1$ is not attractive, i.e. the profit is negative. To satisfy the first condition, we use Eq. (10) and obtain the optimal refund per unit

11 $$\begin{aligned} \beta ^*= \frac{K_2-\alpha q_2-q_2(p_2-v_2)\int _0^Tf_{3 \mathrm {B}_2}y_3 \, \mathrm {d}t}{q_2\int _0^T f_{3 \mathrm {B}_2}y_3 \, \mathrm {d}t}, \end{aligned}$$

where we used that $f_{1 \mathrm {B}_2}=0$ (see Sect. 2). To achieve negative profit for using $\mathrm {B}_1$, we need to choose the parameter $\gamma$ such that developing a broad-spectrum antibiotic $\mathrm {B}_1$ and applying it in compartments $Y_1$ and $Y_3$ (see treatment I in Sect. 2) is not more attractive than developing a narrow-spectrum antibiotic $\mathrm {B}_2$ according to treatment IV (see Sect. 2). Thus, the refunding scheme needs to satisfy

12 $$\begin{aligned} \alpha q_1 + q_1\int _0^{T} \left[ \beta ^*\frac{f_{3\mathrm {B}_1}y_3}{\gamma f_{1\mathrm {B}_1}y_1+f_{3\mathrm {B}_1}y_3} + (p_1-v_1)\right] (f_{1\mathrm {B}_1}y_1+f_{3\mathrm {B}_1}y_3) \, \mathrm {d}t - K_1 < 0.\nonumber \\ \end{aligned}$$

If we evaluate Inequality (12) as an equality, we obtain a critical value for $\gamma$ (i.e., a critical non-resistance penalty), denoted by $\gamma ^*$, for certain values of $\beta ^*$, $p_1$, and $v_1$. For $\gamma >\gamma ^*$, Inequality (12) holds. Inequality (12) together with Eq. (11) imply that it is more profitable to produce a narrow-spectrum antibiotic $\mathrm {B}_2$ and obtain a higher refund than to develop a broad-spectrum antibiotic $\mathrm {B}_1$ and sell more units. We observe that this critical value is uniquely determined, since the left side is strictly decreasing in $\gamma$. We discuss conditions for the existence of $\gamma ^*$ in the next section.

Third, we need to make sure that a narrow-spectrum antibiotic $$\mathrm {B}_2$$ is not used for wild-type bacterial strains (see the 50/50 treatment III in Sect. 2). Since a narrow-spectrum antibiotic may also be effective against wild-type strains, the refunding scheme should exclude any incentives to use $$\mathrm {B}_2$$ in compartment $$Y_1$$. In terms of our refunding scheme, this could be achieved by replacing the 50/50 treatment involving antibiotic $$\mathrm {B}_1$$ on the left-hand side of Eq. (12) with the 50/50 treatment involving antibiotic $$\mathrm {B}_2$$. Note that the resulting critical value for $$\gamma $$, which we denote by $$\gamma ^{**}$$, is different from $$\gamma ^*$$. An alternative to imposing this additional constraint on the refunding scheme is to implement strict medical guidelines which demand that less-effective antibiotics should not be used in compartment $$Y_1$$.

**Fig. 7:**
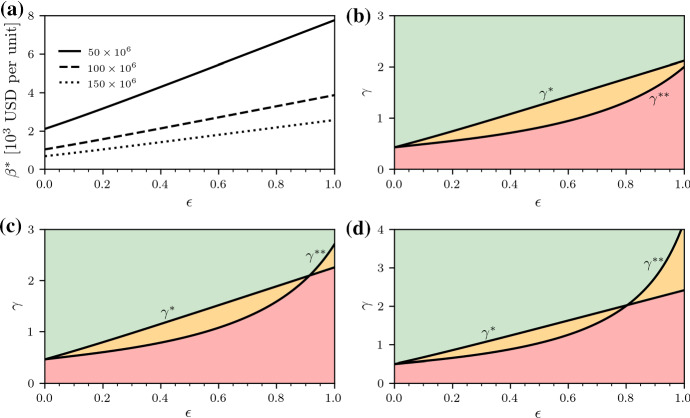
(Color figure online) Critical refunding parameters. (**a**) The critical refund per unit $$\beta ^*$$ increases with $$\epsilon $$ and it decreases with population size. (**b**–**d**) The critical non-resistance penalties $$\gamma ^*$$, $$\gamma ^{**}$$ for different values of $$\epsilon $$ and population sizes $$50\times 10^6$$ (**b**), $$100\times 10^6$$ (**c**), and $$150\times 10^6$$ (**d**). The values of $$\gamma ^*$$ and $$\gamma ^{**}$$ are indicated by the solid black lines. The shown results were obtained by numerically solving Eq. (1) with a classical Runge–Kutta scheme in the time interval [0, *T*] with $$T=100$$ for $$\lambda =100$$, $$d=1$$, $$c=1.5$$, $$b=0.03$$, $$r_{i}=(2-k_i) 0.1$$ ($$k_i$$ is the number of effective antibiotics in the *i*th infected compartment), $$h=1$$, $$s=0.05$$, $$\epsilon \ge 0$$. The initial conditions are $$x(0)=50$$, $$y_1(0)=33.33$$, $$y_2(0)=y_3(0)=y_4(0)=0$$. All compartments were rescaled according to the population sizes shown

Together, Eqs. (11) and (12) determine the refunding scheme that ensures that a pharmaceutical company breaks even at time *T* after developing and effectively using a narrow-spectrum antibiotic, without (primarily) focusing on the development of broad-spectrum antibiotics.

### Numerical Example

We now focus on an example to illustrate how our refunding scheme can incentivize the development of narrow-spectrum antibiotics. For this purpose, we use the parameters listed in the last column of Table 1. To work with reasonable population sizes, we apply our refunding scheme to populations with 50, 100, and 150 million people and rescale the corresponding compartments that we used to determine the antibiotic consumption in Fig. 6.

We first determine the critical refund per unit $$\beta ^*$$ according to Eq. (11) and show the results in Fig. 7 (a). Since the consumption $$C_{\mathrm {B}_2}$$ decreases with $$\epsilon $$ (see Fig. 6), the critical refunding parameter $$\beta ^*$$ has to increase with $$\epsilon $$. Before discussing the corresponding critical broad-spectrum penalties $$\gamma ^*$$ and $$\gamma ^{**}$$, we briefly summarize the conditions for their existence and distinguish three cases.

Case I: If $q_i a+ q_i \int _{0}^T \left[ \beta ^*+(p_i-v_i) \right] (f_{1\mathrm {B}_i}y_1+f_{3\mathrm {B}_i}y_3) \mathrm {d}t - K_i< 0$ ($i=1,2$ and $f_{(\cdot )}$ is chosen according to some treatment protocol), we find that Eq. (12) is satisfied for any $\gamma > 0$, independent of the underlying refunding scheme since, for finite $\gamma$,
  13 $$\begin{aligned} \beta ^*\frac{f_{3\mathrm {B}_i}y_3}{\gamma f_{1\mathrm {B}_i}y_1+f_{3\mathrm {B}_i}y_3}=\beta ^*\frac{1}{1 + \gamma \frac{f_{1\mathrm {B}_i}y_1}{f_{3\mathrm {B}_i}y_3}} < \beta ^*. \end{aligned}$$
Case II: If $q_i a+ q_i \int _{0}^T \left[ \beta ^*+(p_i-v_i) \right] (f_{1\mathrm {B}_i}y_1+f_{3\mathrm {B}_i}y_3) \mathrm {d}t - K_i > 0$ and $q_i a+ q_i \int _{0}^T (p_i-v_i) (f_{1\mathrm {B}_i}y_1+f_{3\mathrm {B}_i}y_3) \mathrm {d}t - K_i < 0$, there exists a $\gamma > 0$ such that the left-hand side of Eq. (12) (for $\mathrm {B}_i$ and corresponding refunding parameters) is equal to zero.
Case III: If $q_i a+ q_i \int _{0}^T (p_i-v_i) (f_{1\mathrm {B}_i}y_1+f_{3\mathrm {B}_i}y_3) \mathrm {d}t - K_i > 0$, it is not possible to satisfy Eq. (12) (for $\mathrm {B}_i$ and corresponding refunding parameters), since $p_i-v_i$ is too large.

For the parameters of Table 1, we show the resulting values of $$\gamma ^*$$ and $$\gamma ^{**}$$ as a function of $$\epsilon $$ in Fig. 7 (b–d). We observe that $$\gamma ^*$$ and $$\gamma ^{* *}$$ always exist for the chosen parameters (case II). Case I does not exist in the outlined example, since $$\beta ^*$$ (Eq. (11)) is large enough. For the chosen values of $$p_i$$ and $$v_i$$, we do not observe case III in Fig. 7 (b–d) either. In real-world applications of our refunding scheme, one can always avoid case III by, for instance, reducing the refunding offset $$\alpha $$.

To summarize:

- For intermediate consumption of $$\mathrm {B}_2$$ in 50/50 treatment (see treatment III in Sect. 2) and corresponding returns, a finite $$\gamma ^{**}$$ exists (see Fig. 7 (b–d)). Within the green-shaded regions of Fig. 7 (b–d), Eq. (12) is satisfied for $$\mathrm {B}_1$$ and $$\mathrm {B}_2$$ ($$\gamma > \gamma ^*$$ and $$\gamma > \gamma ^{**}$$), whereas the left-hand side of Eq. (12) is positive for $$\mathrm {B}_1$$ and $$\mathrm {B}_2$$ within the red-shaded regions ($$\gamma < \gamma ^*$$ and $$\gamma < \gamma ^{**}$$).
- Within the orange-shaded regions of Fig. 7 (b–d), either $\gamma > \gamma ^*$ or $\gamma > \gamma ^*$.
- If the expected return associated with the $\mathrm {B}_2$ treatment III of Sect. 2 is too large, there is no $\gamma >0$ that discourages pharmaceutical companies from developing such drugs.

### Generalizations

We now generalize the refunding scheme of the previous sections to account for possible treatment options with more than two antibiotics. We assume that $N_1$ antibiotics are used currently and that $N_2$ new antibiotics are developed, such that the total number of (potential) antibiotics is $N=N_1+N_2$. For the outlined scenario, the underlying resistance dynamics are described by the general antibiotic resistance model in Appendix A. Note that before new antibiotics are introduced, there is at least 1 non-resistant strain and up to $2^{N_1}-1$ strains that are resistant to some antibiotic. Furthermore, there is one class of bacterial strains that is resistant to all antibiotics currently on the market. The class of microbes that is resistant to all *N* antibiotics has the index ${\hat{k}}=2^N$. The generalized refunding scheme still consists of a fixed refund $\alpha$ and a variable refund that depends on the use of the antibiotic in different compartments. The scaling parameter $\gamma _1\in [0,\infty )$ “punishes” the use of the antibiotic for wild-type strains by decreasing the refund. In addition, $\gamma _2$ scales the reward of the use of the antibiotic for strains that are resistant to some, but not all, antibiotics currently on the market. Note that $\gamma _2$ could be negative, such that the refund still increases in the use for partially resistant strains. Lastly, the refund strongly increases in the use for fully resistant strains in the class ${\hat{k}}=2^N$.

The generalized refunding scheme is given by

14 $$\begin{aligned} g(\tilde{\mathbf {f}_i}) = \beta \frac{\sum _{j=2}^{2^N}f_{j \mathrm {B}_i}y_j}{\gamma _1f_{1 \mathrm {B}_i}y_1+\gamma _2\sum _{j=2}^{2^N-1}f_{j \mathrm {B}_i}y_j + f_{{\hat{k}} \mathrm {B}_i}y_{{\hat{k}}}}, \end{aligned}$$

where $\tilde{\mathbf {f}}_i$ denotes the vector of the usage of a newly developed drug $\mathrm {B}_i$ in all infected compartments $Y_j$ with $j \in \{1,\dots ,N\}$ (notation as defined in Appendix A). The use of antibiotics in infected compartment *j* is $f_{j \mathrm {B}_i}y_j$.

The break-even conditions can be established as for the model with two antibiotics, but now with adjusted total consumption per antibiotic and with the generalized refunding scheme.

Similar to the extension to more than two antibiotics, the refunding scheme can be generalized when more than one pharmaceutical company should be given incentives to pursue R &D on narrow-spectrum antibiotics. In such cases, the refunding parameters have to be adjusted, such that with lower sales volumes for each company, it is still profitable to undertake R &D investments.

## Discussion

Using a refunding scheme as designed above in practice requires a series of additional considerations which we discuss in this section. In particular, the scheme must work under a variety of sources of diagnostic, treatment, and R &D uncertainty. It should also promote the development of the R &D ecosystem and should not discourage the development of broad-spectrum antibiotics if it is impossible to develop a narrow-spectrum antibiotic.

### Multi-dimensional R &D Uncertainties

The development and usage of antibiotics are subject to a variety of uncertainties. In particular, companies may not know at the start of a development process against which type of bacterial strains the drug that might emerge will be effective. Such uncertainties can be taken into account as follows. Suppose a pharmaceutical company starts an R &D process for an antibiotic, but does not know initially whether it will turn out to be broad-spectrum or narrow-spectrum, as this will only become clear during or, in the worst case, at the end of the development process.

A possible solution to this issue is setting the value of the refund per unit, $\beta$, equal to the optimal refund per unit, $\beta ^*$, plus some $\delta >0$ (i.e., $\beta =\beta ^*+ \delta$). This ensures that developing a narrow-spectrum antibiotic produces a small positive expected profit. Moreover, we can ensure that the company breaks even if it develops a broad-spectrum antibiotic by setting $\gamma =\gamma ^*$ for the given value of $\beta ^*+ \delta$.

With these parameters, starting the R &D investment is profitable and the incentives for a narrow-spectrum antibiotic are maximal. If during the R &D process, a narrow-spectrum opportunity emerges, it will be chosen, since expected profits will be higher than for a broad-spectrum antibiotic. However, the company also breaks even for a broad-spectrum antibiotic if such an opportunity emerges. Hence, investing in R &D remains profitable even if it is impossible at the start to evaluate whether a broad-spectrum or narrow-spectrum antibiotic will result from the R &D investment, as such uncertainty does not generate additional profit risk.

### Diagnostic, Treatment, and Usage Uncertainties

The refunding scheme relies on the ability of doctors to rapidly identify the strain of bacteria that caused a certain infection. For a fraction of such treatments, this may be impossible—in particular in emergency situations or when rapid, high-throughput diagnostic devices are unavailable. Yet, certain bacterial strains can already be identified in a few hours by using peptide nucleic acid (PNA) fluorescent in-situ hybridization (FISH) tests, mass spectroscopy, and polymerase chain reaction (PCR)-based methods (Kothari et al. 2014). While traditional, slow, culture-based identification techniques are still very common, rapid diagnostics are available in major medical centers in the USA.

New diagnostic approaches are currently developed by several companies to significantly improve the speed and comprehensiveness of diagnostics of pathogens that cause a disease. These approaches are based on sequencing a targeted part of the DNA/RNA of an infected patient. Then, with machine learning tools, the sequenced data are analyzed and a diagnostic report on the pathogen causing the infection is produced. The aim is to have a diagnosis within less than 24 hours. At the moment, test kits are already available for some multi-drug resistant bacteria (see, e.g., https://clemedi.com/products2/tuberculosis/) and regulatory approval is expected this year or the next.

Nevertheless, it will take quite some time to use such tools at a greater scale and to spread the technology. A refunding scheme would significantly help to accelerate development and diffusion, since having comprehensive and rapid diagnostic tools will be in the interest of pharmaceutical companies developing new antibiotics against resistant bacteria.

Refunding schemes can be readily adapted to allow for diagnostic and treatment uncertainties. For instance, one could base refunding only on diagnosed strains of bacteria against which newly developed antibiotics are used. The refunding parameters have to be adapted accordingly. Basing refunding only on those cases in which the bacterial strain has been diagnosed and reported would provide further (direct and indirect) incentives for biotech companies to develop fast diagnostic tests that help identify the sources of infections.

A further refinement would be to provide a refund in case a newly developed antibiotic is used and turns out to be effective. Such success targeting would be desirable, but may not be easily implementable in practice. As long as the success rates of an antibiotic that is effective against particular bacterial strains are known or can be estimated with sufficient precision, collecting the usage and bacterial strain data would be sufficient to provide desirable incentives to engage in R &D for narrow-spectrum antibiotics.

### Small Firms and the R &D Ecosystem

Both small biotech companies and large pharmaceutical companies play a significant role in developing new antibiotics. The flexibility, nimbleness, and flat organizational structure of smaller biotech companies that specialize in innovative antibacterial treatments can be very effective for the development of new antibiotics. Therefore, while refunding will mostly benefit large pharmaceutical companies, the anticipation of such refunds is expected to also motivate smaller biotech companies to increase their R &D efforts. These smaller companies can expect significant rewards when they sell or license their patents to larger companies. Moreover, in the presence of a refunding mechanism, small biotech companies may receive much more start-up funding both from venture capitalists^8^ and larger pharmaceutical companies, and one might even consider using the antibiotics fund for this purpose as well, e.g. by co-funding business incubators. Hence, it is expected that the refunding scheme will be nourishing for the entire ecosystem that develops new antibiotics. A significant literature has documented the importance of dynamic, open R &D ecosystems for innovation (see, e.g., Shaikh and Levina 2019; Cohen et al. 2019) and assessed how policy initiatives can nourish such systems (Audretsch et al. 2020).

### The Antibiotics Fund, Differentiating Fees, and Participating Countries

A necessary condition for the functioning of our refunding scheme is the existence of an antibiotics fund with sufficient equity to cover R &D incentives. Similar to the recently established AMR Action Fund, which aims at bridging the gap between the pipeline for innovative antibiotics and patients, an antibiotics fund should be started by initial contributions from industry and public institutions. Since it is in the collective self-interest of the pharmaceutical industry to solve the antibiotics dilemma—as otherwise, many other business lines and their reputation will be harmed—, a significant contribution from the industry can be expected to set up the antibiotics fund, as it was the case for the AMR Action Fund. In addition, a continuous refilling of the fund can be achieved by levying a fee (or Pigouvian tax, see Hollis and Maybarduk 2015) on every use of existing antibiotics. These fees have to be set in such a way that the antibiotics fund will never be empty. Since the (sometimes excessive) use of existing antibiotics (e.g., in agricultural settings as described in the Seventh Report of the Committee on Science and Technology 1998; Casey et al. 2013; Xu et al. 2020) is a major driver of today’s AMR crisis, levying fees will not only help to continuously refill the fund, but it may also help to use existing antibiotics more cautiously. The fees could be set differently for each antibiotic and depend on the risk of generating resistance. Moreover, in order to provide equal access to antibiotics for humans, fees could be levied mostly on non-human use of antibiotics. Ultimately, both the antibiotics fund and refunding scheme provide mechanisms to internalize the externality in antibiotics use, namely the generation of resistant bacteria, without compromising universal access to antibiotics for humans.

As in the context of slowing down climate change, the ideal implementation would involve a global refunding scheme administered by an international agency, because reducing resistance is a global public good. However, also similar to implementing climate change policies, worldwide adoption is expected to be extremely difficult and might be impossible to achieve. As a starting point, a set of industrialized countries should agree to a treaty that fails if any of them does not participate. Once an antibiotic fund has been initiated, a treaty should establish the continuing financing of the antibiotics fund and the refunding scheme. The gains would be large and may lead to long-standing self-enforcing incentives to substantially and continuously increase the chances to develop antibiotics against resistant bacteria. If attempts to build a larger coalition fail, the European Union or the USA could take the lead and become the first country, or coalition of countries, that implements a refunding scheme for antibiotics.

### Charging High Prices for Antibiotic Use

One could also achieve sufficiently strong incentives to develop new antibiotics without a refunding scheme, by allowing for very high prices when an antibiotic is used against bacterial strains that are resistant against other antibiotics. We do not pursue this approach, since enormously high prices for a treatment would raise ethical and health concerns. For instance, with high prices, certain therapies might then only be affordable to high-income households. Moreover, many infections by bacteria strains may not be treated appropriately and this may fuel the spreading of resistant germs.

## Conclusions

The rapid rise of antibiotic resistance is a serious threat for global public health. No new major class of antibiotics has been registered or patented for more than three decades (see Fig. 1). One important cause for this stagnation in antibiotic R &D is that the antibiotics market is broken (Böttcher et al. 2022b). Antibiotic R &D is regarded as risky and less profitable than other pharmaceutical R &D options. A key issue underlying the reluctance of pharmaceutical companies to invest in antibiotic R &D is that new antibiotic classes should be used scarcely to limit the emergence of de novo resistance. To reduce risks associated with antibiotic R &D, the development process could be supported with (i) additional funding and push and pull incentives and (ii) support for basic research and innovation.

We introduced a new framework to mathematically describe the emergence of antibiotic resistance in a population that is treated with *n* antibiotics. We then used this framework to develop a market-based refunding scheme that can solve the antibiotics dilemma. That is, it can incentivize pharmaceutical companies to reallocate resources to antimicrobial drug discovery and, in particular, to the development of narrow-spectrum antibiotics that are effective against multiresistant bacterial strains. We describe how such a refunding scheme can cope with various sources of uncertainty inherent to R &D for antibiotics, as well as with diagnostic and treatment uncertainties.

Our study opens up several avenues for future research. One worthwhile direction for future work is to combine our methods with control theory (Chehrazi et al. 2019; Xia et al. 2021; Asikis et al. 2022; Böttcher et al. 2022a) to study how many new antibiotics are needed on average in a certain time interval (e.g., 10–20 years) to create a stable supply of effective treatment options and to keep the emergence of antibiotic resistance at a minimum. Another important direction is to estimate the minimum size of the proposed funding scheme for different regions to make antibiotic R &D viable under current and/or modified market conditions.

## Figures and Tables

**Fig. 8:**
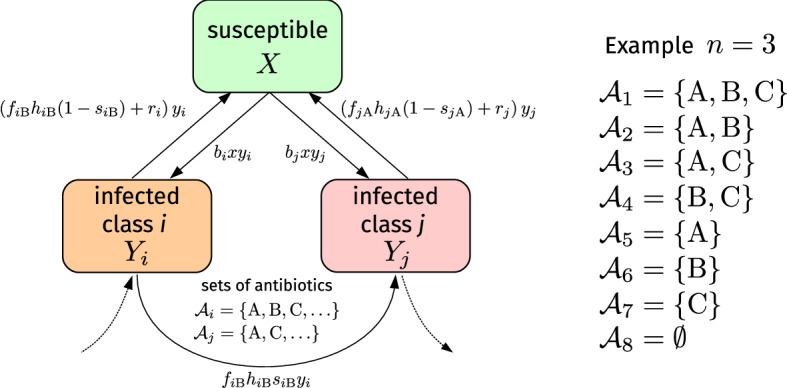
(Color figure online) Model schematic. Susceptible individuals (i.e., individuals in state *X*) can be infected by individuals in state $Y_i$ (i.e., individuals who are infected with bacterial strain *i*) at rate $b_i$. Infected individuals in state $Y_i$ recover spontaneously at rate $r_i$. The effective antibiotic-induced recovery rate associated with individuals in state $Y_i$ and antibiotic $\mathrm {B}$ is $f_{i\mathrm {B}} h_{i\mathrm {B}}$. Only a fraction $1-s_{i\mathrm {B}}$ of individuals in state $Y_i$ recovers after a treatment with antibiotic $\mathrm {B}$. The remaining fraction $s_{i\mathrm {B}}$ becomes resistant against antibiotic $\mathrm {B}$ and ends up in a compartment $Y_j$ of bacterial strains exhibiting more resistances. The sets of effective antibiotics in compartments $Y_i$ and $Y_j$ are ${\mathcal {A}}_i$ and ${\mathcal {A}}_j$, respectively. Infection and recovery processes with the respective rates are also present in compartment $Y_j$. For $n=3$ antibiotics, we show the possible antibiotic-treatment classes ${\mathcal {A}}_1,{\mathcal {A}}_2,\dots ,{\mathcal {A}}_8$. We account for birth and death dynamics in the antibiotic resistance model (A1) (not shown in the model schematic for conciseness)

**Fig. 9:**
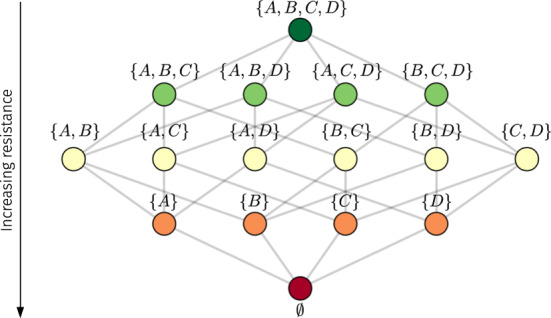
(Color figure online) Antibiotic resistance network. For $n=4$ antibiotics, we show the corresponding antibiotic resistance network. Nodes represent states in which the indicated antibiotics are effective and edges between nodes represent the development of resistant strains due to the usage of certain antibiotics. In the example displayed, single-antibiotic therapy is being used. That is, no combinations of antibiotics are being administered to patients

**Fig. 10:**
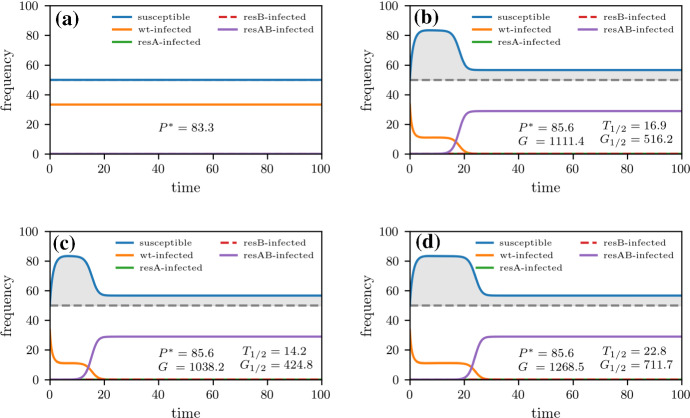
(Color figure online) Treatment with two antibiotics. We numerically solve Eqs. (B1) and (B3) with a classical Runge–Kutta scheme for $\lambda =100$, $d=1$, $c=1.5$, $b=0.03$, $r_{1}=0$, $r_{2}=r_{3}=0.1$, $r_{4}=0.2$. In panel (**a**), we set $h=0$ (i.e., no treatment) and $h=1$ in the remaining panels. Panel (**b**) shows a solution of Eq. (B1) (“single-antibiotic therapy”). Panels (**c**) and (**d**) show solutions of Eq. (B3) (“combination therapy”) with $q=10^{-5}>s^2=10^{-6}$ and $q=10^{-8}<s^2=10^{-6}$, respectively. If $q>s^2$, the gain is smaller for multiple treatment. We use $P^*$ and *G* to indicate the total stationary population size (see Eqs. (2) and (B2)) and gain of uninfected in the considered time interval (see Eq. (3)), respectively. The gain *G* corresponds to the gray-shaded region and the characteristic resistance time scale $T_{1/2}$ is the time when the proportion of completely resistant strains is 50% (see Eq. (4)). $G_{1/2}$ is the gain in the time interval $[0,T_{1/2}]$. The initial conditions are $x(0)=50$, $y_1(0)=33.33$, and $y_2(0)=y_3(0)=y_4(0)=0$. Note that the proportions of cases with A-resistant strains (solid green line) and B-resistant strains (dashed red line) are zero in (**a,b,c**) and very small in (**b**)

**Fig. 11:**
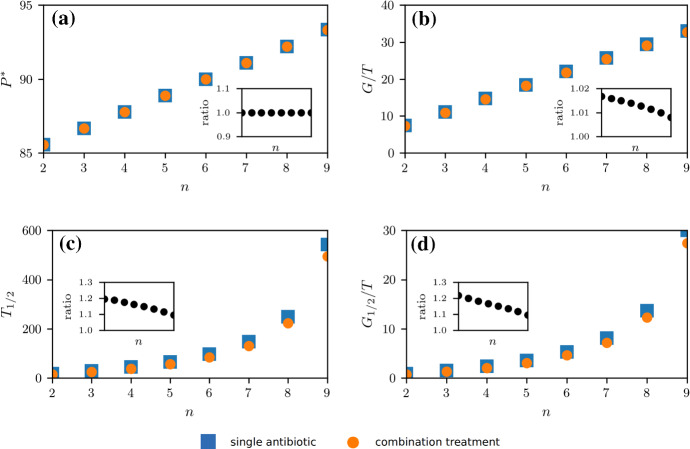
(Color figure online) Symmetric treatment with multiple antibiotics. We numerically solve the *n*-antibiotic generalizations of Eqs. (B1) and (B3) with a classical Runge–Kutta scheme in the time interval [0, *T*] with $T=600$ and $\lambda =100$, $d=1$, $c=1.5$, $b=0.03$, $r_{i}=(n-k_i) 0.1$ ($k_i$ is the number of effective antibiotics in the *i*th infected compartment), $h=1$, $s=10^{-3}$, and $q=10^{-2.5 n}$. Panels (**a**) and (**b**) show the total stationary population size $P^*$ (see Eqs. (2) and (B2)) and the gain of uninfected individuals, *G* (see Eq. (3)), respectively. Panels (**c**) and (**d**) show the time $T_{1/2}$ when the proportion of completely resistant strains is 50% (see Eq. (4)) and the gain $G_{1/2}$ in the time interval $[0,T_{1/2}]$, respectively. The insets in each panel show the ratio of the single and combination therapy values. The initial conditions are $x(0)=50$, $y_1(0)=33.33$, and $y_2(0)=y_3(0)=y_4(0)=0$

## Data Availability

All data, materials, and codes used are available on request.

## Appendix A: Modeling Antibiotic Treatment with *n* Antibiotics

In this appendix, we formulate a mathematical framework to model antibiotic resistance dynamics with *n* antibiotics. Our framework is able to account for an arbitrary number of different antibiotics, while previous models (Uecker and Bonhoeffer 2021; Bonhoeffer et al. 1997; Levin and Bonten 2004) only considered two to three distinct antibiotics and compared different treatment protocols such as temporal variation and combination therapy. Similar “low-dimensional” descriptions of antibiotic resistance have been used to study the economic problem of optimal antibiotic use (Laxminarayan and Brown 2001).

As in the main text, we describe the interaction between infectious and susceptible individuals using an SIS-type model whose infected compartment is sub-divided into compartments such that each can be treated with certain antibiotics. We indicate a susceptible state by *X* and use $Y_i$ to denote infected states that are sensitive to antibiotics in the set ${\mathcal {A}}_i$. If the set ${\mathcal {A}}_2$ contains two antibiotics $\mathrm {A}$ and $\mathrm {B}$ (i.e., ${\mathcal {A}}_2=\{\mathrm {A},\mathrm {B}\}$), individuals in state $Y_2$ can be treated with these two antibiotics but not with a potentially available third antibiotic $\mathrm {C}$ that can be used to treat individuals in state $Y_1$, where ${\mathcal {A}}_1=\{\mathrm {A},\mathrm {B},\mathrm {C}\}$ (see Fig. 8).

The rate equations of our general resistance dynamics model are

A1 $$\begin{aligned} \begin{aligned} \frac{\mathrm {d} x}{\mathrm {d} t}&=-x\sum _{i=1}^N b_i y_i+\sum _{i=1}^{N}r_i y_i + \left[ \sum _{i=1}^N \sum _{j\in {\mathcal {A}}_i} f_{i j} h_{ij} \left( 1-s_{ij}\right) y_i\right] +\lambda - d x, \\ \frac{\mathrm {d} y_i}{\mathrm {d} t}&=b_i x y_i - r_i y_i-c_i y_i - \sum _{j\in {\mathcal {A}}_i} f_{i j} h_{ij} y_i+\sum _{k < i} \sum _{j\in {\mathcal {S}}({\mathcal {A}}_i)} f_{kj} h_{kj} s_{kj} y_k, \end{aligned} \end{aligned}$$

where *x* and $y_i$ denote the proportions of individuals in states *X* and $Y_i$, respectively. The birth rate of new susceptible individuals is $\lambda$ and the corresponding death rate is *d*. An infection with bacterial strain *i* occurs at rate $b_i$. Additional resistance mechanisms (e.g., horizontal gene transfer as studied by Reygaert 2018; Sun et al. 2019) may be modeled via spontaneous transitions of certain proportions of the population from state $Y_i$ to $Y_j$ ($j>i$). We denote the (spontaneous) recovery rate by $r_i$, and we use the convention that as *i* increases the corresponding bacteria become more resistant. We also account for the fitness cost associated with antibiotic resistance (i.e., $r_{i}-r_{1} >0$ for all $i \in \{2,\dots ,N\}$) as indicated in Andersson (2006). Antibiotic-induced recovery from compartment *i* with antibiotic $j\in {\mathcal {A}}_i$ occurs at rate $h_{ij}$. The quantity $f_{i j}$ is the proportion of antibiotic $j\in {\mathcal {A}}_i$, relative to other antibiotics, that is used to treat individuals in state $Y_i$. However, only a fraction $1-s_{ij}$ of individuals treated with antibiotic *j* recovers, whereas the remaining fraction $s_{ij}$ becomes resistant to antibiotic $j\in {\mathcal {A}}_i$. For infected individuals in state $Y_i$, the death rate is $c_i$. The set ${\mathcal {S}}({\mathcal {A}}_i)$ contains all antibiotics that were used to arrive at a partially or completely resistant compartment $Y_i$ from other states $Y_k$ ($k<i$) with fewer resistances. For example, the use of single antibiotics (i.e., one per patient) is described by ${\mathcal {S}}({\mathcal {A}}_i)={\mathcal {A}}_1\setminus {\mathcal {A}}_i$.

If antibiotics that are available to treat patients in state $Y_i$ are administered uniformly, the values of $f_{ij}$ are $1/|{\mathcal {A}}_i|$, where $|{\mathcal {A}}|$ is the cardinality of the set ${\mathcal {A}}$. We show an example of a corresponding antibiotic resistance network for $n=4$ antibiotics in Fig. 9. Nodes in such a resistance network represent states $Y_i$ and edges describe treatment pathways. In the example we show in Fig. 9, only single antibiotics (no combinations) are being used for treatment.

The general antibiotic resistance model (see Eq. (A1)) has *N* different compartments, which correspond to *N* resistance states, each accounting for a certain set of effective antibiotics. We denote the total number of antibiotics by *n*. What is the number of resistance states *N* that belongs to a certain number of antibiotics *n*? Considering the antibiotic resistance network of Fig. 9, we observe that the total number of resistance states *N* is the sum over all possible combinations of single antibiotics plus one (representing the completely resistant state). For *n* different antibiotics, we thus have to consider $$N=1+\sum _{k=1}^n \left( {\begin{array}{c}n\\ k\end{array}}\right) =2^n$$ different elements $Y_i$ ($i\in \{1,2,\dots ,N\}$) of the power set of the set of all antibiotics. We order them in the following way. We denote by $Y_1$ the infected state that can be successfully treated with all antibiotics, while $Y_N$ represents the state in which a person has been infected with a completely resistant strain. Let $k\le n$ be the number of effective antibiotics. For a wild-type strain, the number of effective antibiotics is $k=n$. In each layer of the antibiotic resistance network, there are $$\left( {\begin{array}{c}n\\ k\end{array}}\right) $$ different strains. For a treatment with single antibiotics (see Fig. 9), there are always $$\left( {\begin{array}{c}n\\ k\end{array}}\right) $$ nodes with *k* edges in a certain layer that need to be connected to $$\left( {\begin{array}{c}n\\ k-1\end{array}}\right) $$ nodes in the following layer. Using the relation

A2 $$\begin{aligned} \frac{k \left( {\begin{array}{c}n\\ k\end{array}}\right) }{\left( {\begin{array}{c}n\\ k-1\end{array}}\right) }=\frac{(n-k+1)\,!}{(n-k)\,!}=n-k+1 \end{aligned}$$

shows that $n-k+1$ nodes from the current layer are mapped to one node in the next layer. In the first layer ($k=4$) of the network with $n=4$ that we show in Fig. 9, one node from the current layer is mapped to one node in the next layer. In the second layer ($k=3$), two nodes are mapped to one node in the third layer. Similar considerations apply to other treatment protocols (e.g., combination treatment with multiple antibiotics) and help to formulate the corresponding set of rate equations.

Previous models of antibiotic resistance only considered the treatment with two and three antibiotics (Bonhoeffer et al. 1997; Levin and Bonten 2004; Day and Gandon 2012; Uecker and Bonhoeffer 2021). Our generalization to *N* compartments allows us to provide insights into the higher-dimensional nature of the dynamical development of antibiotic resistance. In Appendices B and C, we compare the outlined single-antibiotic therapy approach with combination treatment for different numbers of antibiotics. We also demonstrate in Appendix C that the mathematical form of the stationary solution of Eq. (A1) is unaffected by the number of antibiotics. Still, more antibiotics can be useful to slow down the development of completely resistant strains, suggesting that rolling out more antibiotics is useful (see Appendix C). However, as we discuss in the main text, fostering the development of particular types of narrow-spectrum antibiotics is much more powerful than developing broad-spectrum antibiotics to slow down the occurrence of completely resistant strains and to reduce the number of deaths.

## Appendix B: Combination Therapy Versus Targeted Use of Antibiotics

To illustrate the difference between combination- and single-antibiotic therapy, we first derive the corresponding mathematical results for $n=2$ antibiotics $\{\mathrm {A},\mathrm {B}\}$ (Bonhoeffer et al. 1997). The case where $n>2$ is discussed in Appendix C.

For $n=2$ antibiotics, the corresponding sets of antibiotics for the $N=4$ infected compartments are $A_1=\{\mathrm {A},\mathrm {B}\}$, $A_2=\{\mathrm {A}\}$, $A_3=\{\mathrm {B}\}$, and $A_4=\emptyset$. Based on Eq. (A1), the treatment of patients with single broad-spectrum antibiotics can be described by:

B1 $$\begin{aligned} \frac{\mathrm {d} x}{\mathrm {d} t}&=-b x \left( y_1+y_2+y_3+y_4\right) +r_1 y_1+r_2 y_2 + r_3 y_3+r_4 y_4 \nonumber \\&+ h (1-s) \left( y_1+y_2+y_3\right) +\lambda - d x,\nonumber \\ \frac{\mathrm {d} y_1}{\mathrm {d} t}&=\left( b x - r_1 - h-c \right) y_1, \nonumber \\ \frac{\mathrm {d} y_2}{\mathrm {d} t}&=\left( b x - r_2 - h-c \right) y_2+\frac{1}{2}h s y_1, \\ \frac{\mathrm {d} y_3}{\mathrm {d} t}&=\left( b x - r_3- h-c \right) y_3+\frac{1}{2}h s y_1, \nonumber \\ \frac{\mathrm {d} y_4}{\mathrm {d} t}&=\left( b x - r_4-c \right) y_4 +h s \left( y_2 +y_3\right) ,\nonumber \end{aligned}$$

where we set $c_j=c$, $b_{ij}=b$, $s_{ij}=s$, $h_{ij}=h$, and $f_{1 \mathrm {A}}=f_{1 \mathrm {B}}=1/2$, $f_{1 \mathrm {A B}}=0$, $f_{2 \mathrm {A}}=1$, $f_{2 \mathrm {B}}=f_{2 \mathrm {AB}}=0$, $f_{3 \mathrm {B}}=1$, and $f_{3 \mathrm {A}}=f_{3 \mathrm {AB}}=0$.

In the absence of treatment, the total stationary population is

B2 $$\begin{aligned} P^*=x^*+y_1^*=\frac{r_1+c}{b}+\frac{\lambda }{c}-\frac{d}{b}-\frac{d r_1}{c b}. \end{aligned}$$

Analytical expressions for *G* and $T_{1/2}$ for some specific parameter choices are summarized in Bonhoeffer et al. (1997). We use the set of equations

B3 $$\begin{aligned} \frac{\mathrm {d} x}{\mathrm {d} t}&=-b x \left( y_1+y_2+y_3+y_4\right) +r_1 y_1+r_2 y_2 + r_3 y_3+r_4 y_4 \nonumber \\&\quad + h (1-q) y_1+ h (1-s) \left( y_2+y_3\right) +\lambda - d x,\nonumber \\ \frac{\mathrm {d} y_1}{\mathrm {d} t}&=\left( b x - r_1 - h-c\right) y_1, \nonumber \\ \frac{\mathrm {d} y_2}{\mathrm {d} t}&=\left( b x - r_2 - h -c\right) y_2, \nonumber \\ \frac{\mathrm {d} y_3}{\mathrm {d} t}&=\left( b x - r_3 - h-c \right) y_3, \nonumber \\ \frac{\mathrm {d} y_4}{\mathrm {d} t}&=\left( b x - r_4-c\right) y_4 +h q y_1+h s \left( y_2 +y_3\right) , \end{aligned}$$

to compare single-antibiotic therapy and targeted use of antibiotics (see Eq. (B1)) with a broad-spectrum treatment that uses combinations of antibiotics $\mathrm {A}$ and $\mathrm {B}$. In this model, we have $s_{1\mathrm{AB}}= q$ and $s_{i j}= s$ otherwise. The parameter *q* is the fraction of double resistances that develop from the combined treatment of the wild-type strain ($Y_1$) with antibiotics $\mathrm {A}$ and $\mathrm {B}$. In Eq. (B3), we set $f_{1 \mathrm {AB}}=1$, $f_{1 \mathrm {A}}=f_{1 \mathrm {B}}=0$, $f_{2 \mathrm {A B}}=1$, $f_{2 \mathrm {A}}=f_{2 \mathrm {B}}=0$, $f_{3 \mathrm {AB}}=1$, and $f_{3 \mathrm {A}}=f_{3 \mathrm {B}}=0$.

We show a comparison between the outlined single-antibiotic and the combination therapy treatment in Fig. 10. If $q>s^2$, we find that, in agreement with earlier results (Bonhoeffer et al. 1997), single-antibiotic treatment outperforms combination therapy. For $q < s^2$ (i.e., for very small probabilities of double resistance resulting from combination treatment of wild-type strains), single-antibiotic treatment is not as efficient as broad-spectrum therapy anymore.

## Appendix C: Properties of the General Model

In this appendix, we establish several properties of the general antibiotic-treatment model (see Eq. (A1)). In particular, in the absence of antibiotic treatment and for sufficiently strong treatment, we show that the mathematical structure of the equation describing the stationary population $P^*$ is unaffected by the number of antibiotics and differences in treatment protocols. The term “sufficiently strong treatment” (Bonhoeffer et al. 1997) means that the growth factor $b/(r_N+c_N)$ in the completely resistant compartment is larger than the growth factor $b/(r_i+c_i+\sum _{j\in A_i} f_{i j} h_{ij})$ in any other compartment $y_i$ ($i < N$). In the absence of antibiotic therapy (i.e., $h_{ij}=0$ for all *i*, *j*), we find that the stationary population of susceptible individuals is $x^* = (r_1+c_1)/b$ and $y^* = \lambda /c_1 - d/b - (d r_1)/(b c_1)$.

The stationary solution under sufficiently strong treatment with $y_N^*\ne 0$ implies that $y_1^*=y_2^*= \cdots = y_{N-1}^*=0$. The corresponding stationary proportions of susceptible and fully resistant individuals are $x^*=(r_N+c_N)/b_N$ and $y_N^*= \lambda /c_N-d/b_N-(d r_N)/(b_N c_N)$, respectively. Even if the stationary solution for a general number of antibiotics *n* has a similar mathematical structure as the solution for $n=2$ antibiotics, the dynamical features of the infected compartments and corresponding characteristics such as $T_{1/2}$ exhibit a more complex dependence on *n*, which we analyze numerically in Fig. 11.

We observe that for the considered parameters in the “$q > s^n$ regime” (see Appendix B), single-antibiotic therapy still outperforms combination treatment. However, the larger the number of antibiotics *n*, the smaller are the relative differences between both the two treatment protocols in terms of the studied performance metrics (see insets in Fig. 11).

## Footnotes

1 For a historical account on the development of antibiotics (see Kingston 2000).

2 https://amractionfund.com/, retrieved on July 13, 2020.

3 Phase III clinical trials are the last phase of clinical research. They have to be successfully completed before regulatory agencies will approve a new drug. Such trials usually involve large patient groups (ca. 300–3000 volunteers who have the disease or condition) and require comparatively long observation periods ranging between 1 and 4 years (FDA 2018b).

4 An alternative mathematical definition of “narrow” and “broad” is to classify combination treatment as a broad-spectrum approach and single-antibiotic therapy as a narrow-spectrum treatment.

5 For the sake of comparability with the results presented by Bonhoeffer et al. (1997), we interpret *x* and $y_i$ as absolute frequencies and not as proportions in our simulations.

6 The refunding scheme is flexible and can incorporate a risk premium to make R &D attractive for risk-averse decision makers. We later extend the refunding scheme to a generalized version that accounts for multiple companies pondering about R &D investments.

7 The fee levied for financing refunds are added to obtain the price paid by users of antibiotics. For simplicity, we assume that the use of antibiotics remains the same with an additional fee. The refunding scheme can easily be adapted to take into account a reduction in antibiotic use when fees are levied. Fees would be levied on non-human use of antibiotics.

8 The global venture capital investment in antimicrobial R &D in the period between 2004 and 2013 amounted to about 3.61 billion USD (Renwick et al. 2016), which is not enough to fund a diverse global AMR drug discovery landscape (DiMasi et al. 2016; Årdal 2020). In this period, 1.6 billion USD of the mentioned total venture capital was invested in the development of broad-spectrum agents, and 446 million USD and 1.56 billion USD were invested in R &D on Gram-negative and Gram-positive antibiotics, respectively.
